# 1381. Do Gut Microbiome Profiles Correlate with Hospital Length of Stay During Hematopoietic Stem Cell Transplantation?

**DOI:** 10.1093/ofid/ofab466.1573

**Published:** 2021-12-04

**Authors:** Angelico Mendy, Kitty Tierney, Tara Mink, Walaa Hussein, Peter Monaco, Claire Weinstein, Prathyusha Kandala, Racheal Wilkinson, Kavya Patel, Senu Apewokin

**Affiliations:** 1 University of Cincinnati, Cincinnati, Ohio; 2 The Jewish Hospital, Cincinnati, Ohio; 3 Emory University, Los Angeles, California; 4 University of Cincinnati Medical Center, Cincinnati, Ohio

## Abstract

**Background:**

Length of stay is not only an indicator of how successful a hospitalized patient’s treatment and recovery is, but is also an indicator of fiscal costs to the hospital. Hematopoietic stem cell transplants (HSCT) patients typically experience extended hospital admissions that can vary significantly patient to patient with hospital discharge dependent upon a recovered white blood cell count. Recent literature suggests a gut microbial influence on hematopoiesis. We sought to explore potential associations between gut microbiome diversity and the length of stay in patients undergoing HSCT in the inpatient setting.

**Methods:**

Within two healthcare systems, we identified patients who would receive conditioning chemotherapy and subsequent HSCT in the inpatient setting. Pre-chemotherapy stool was collected, sequenced with shotgun metagenomics, and analyzed for gut microbial diversity using Inverse-Simpson index. The length of admission or length of stay during their transplant process was recorded. We assessed whether there was an association with gut microbial diversity and length of stay.

**Results:**

24 patients we evaluated for diversity and length of stay. There was no significant correlation between age or gender and length of stay. Significant difference in length of stay was seen between allogenic vs autogenic transplants (p value ≤0.01). Within the 24 patients, lengths of stay ranged from 8 to 36 days with a mean average of 20.9 days. Gut diversity ranged from 1.8 to 23.9. An overall negative association between length of stay and diversity was seen, though this was determined not statistically significant (p value 0.09).

Length of Stay correlation with pre-chemotherapy Gut Microbiome diversity

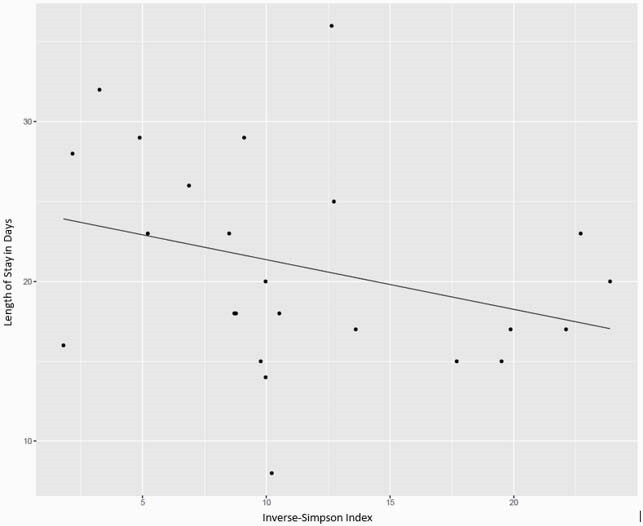

**Conclusion:**

Our study showed no significant association between gut microbial diversity and inpatient length of stay during HSCT. Overall, a trend towards increased length of stay in patients with decreased diversity was noted. Additional studies of greater participant size are necessary to confirm or further study these findings.

**Disclosures:**

**All Authors**: No reported disclosures

